# Systemic T-helper and T-regulatory cell type cytokine responses in rhinovirus vs. respiratory syncytial virus induced early wheezing: an observational study

**DOI:** 10.1186/1465-9921-10-85

**Published:** 2009-09-25

**Authors:** Tuomas Jartti, Maria Paul-Anttila, Pasi Lehtinen, Vilhelmiina Parikka, Tytti Vuorinen, Olli Simell, Olli Ruuskanen

**Affiliations:** 1 Department of Pediatrics, Turku University Hospital, Turku, Finland; 2 Department of Virology, University of Turku, Turku, Finland

## Abstract

**Background:**

Rhinovirus (RV) associated early wheezing has been recognized as an independent risk factor for asthma. The risk is more important than that associated with respiratory syncytial virus (RSV) disease. No comparative data are available on the immune responses of these diseases.

**Objective:**

To compare T-helper_1 _(Th_1_), Th_2 _and T-regulatory (T_reg_) cell type cytokine responses between RV and RSV induced early wheezing.

**Methods:**

Systemic Th_1_-type (interferon [IFN] -gamma, interleukin [IL] -2, IL-12), Th_2_-type (IL-4, IL-5, IL-13) and T_reg_-type (IL-10) cytokine responses were studied from acute and convalescence phase serum samples of sole RV (n = 23) and RSV affected hospitalized wheezing children (n = 27). The pre-defined inclusion criteria were age of 3-35 months and first or second wheezing episode. Analysis was adjusted for baseline differences. Asymptomatic children with comparable demographics (n = 11) served as controls for RV-group.

**Results:**

RV-group was older and had more atopic characteristics than RSV-group. At acute phase, RV-group had higher (fold change) IL-13 (39-fold), IL-12 (7.5-fold), IFN-gamma (6.0-fold) and IL-5 (2.8-fold) concentrations than RSV-group and higher IFN-gamma (27-fold), IL-2 (8.9-fold), IL-5 (5.6-fold) and IL-10 (2.6-fold) than the controls. 2-3 weeks later, RV-group had higher IFN-gamma (>100-fold), IL-13 (33-fold) and IL-10 (6.5-fold) concentrations than RSV-group and higher IFN-gamma (15-fold) and IL-2 (9.4-fold) than the controls. IL-10 levels were higher in acute phase compared to convalescence phase in both infections (p < 0.05 for all).

**Conclusion:**

Our results support a hypothesis that RV is likely to trigger wheezing mainly in children with a predisposition. IL-10 may have important regulatory function in acute viral wheeze.

## Background

Rhinovirus (RV) is the principal pathogen responsible for the common cold. It is also the most common virus being associated with asthma attacks in children (up to 60% of cases) [1,2]. In wheezy children less than 2 years old at emergency room and hospital settings, RV is also a common agent (up to 41-47%; depends on the risk factors of asthma) only second to respiratory syncytial virus (RSV, up to 68% of cases) [3,4]. Studies how viral etiology of early wheezing may contribute to later development of asthma have focused almost exclusively on RSV, but recent studies suggest that RV is equal [5], or more important viral risk factor than RSV [6-8].

Rhinoviruses belong to the *Picornaviridae *family, small non-enveloped viruses containing a single-stranded RNA genome. At least 101 different RV serotypes and over 150 different RV strains have been identified thus far, establishing RVs as the most diverse group of *Picornaviridae *[9,10]. Based on receptor binding properties, RVs are divided into two classes: the major group binding to intracellular adhesion molecule-1 and the minor group binding to the very low density lipoprotein receptors. After viral uptake, RVs trigger cytokine and chemokine responses upon infection that may lead to airway illness.

Many questions remain unanswered regarding the key inflammatory mediators involved in early wheezing episodes associated with RV infection. Despite many *in vitro *studies, the number of *in vivo *studies is very limited and focussed almost exclusively on adult subjects [11-17]. Cytokine gene polymorphism studies are increasingly reported in wheezing children [18-21], but there are no studies comparing cytokine responses in RV and RSV affected young wheezing children. The available comparative data among young children with wheezing is limited to atopic characteristics, which have been more pronounced in RV than RSV affected children [5,22], and thereby, as many studies in adults and one in children, suggest possible role for cytokines involved in T cell differentiation [11-17]. The aim of our observational study was to compare systemic T-helper_1 _(Th_1_), Th_2 _and T-regulatory (T_reg_) cell type cytokine responses between children with RV and RSV induced early wheezing. We hypothesised that RV affected young wheezing children have different T cell cytokine profile compared to RSV affected children.

## Methods

### Subjects

**Figure 1:**
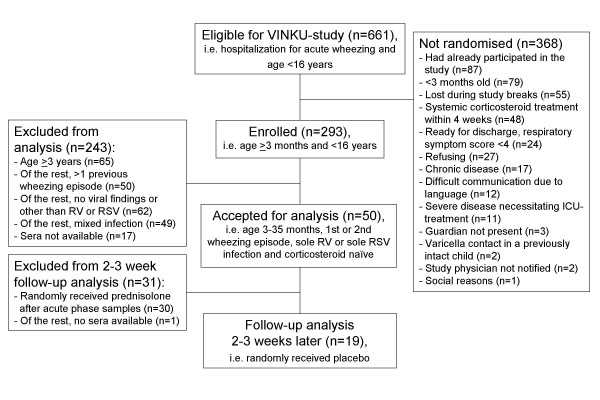
**Study flow chart**.

The study is a substudy of the VINKU study which took place in the Department of Pediatrics of Turku University Hospital (9/2000-5/2002). The original aim was to study the efficacy of oral prednisolone treatment in hospitalized wheezing children in relation to viral etiology, i.e. a half of the patients were randomized to receive oral prednisolon for 3 days and the other half placebo in a double blind design. The methods have been described earlier [7,23]. The present study included all children of the VINKU study who were 3 to 35 months old, had their first or second wheezing episode, had sole RV or RSV infection and had either acute or convalescent phase serum available (Fig. 1). In addition, we included asymptomatic control children who participated to VINKU2 study in the same institution (10/2008-5/2009). They had not had respiratory symptoms within 2 weeks, had never wheezed and had no chronic illnesses other than possible atopy. All recruited control children were included to this analysis. The study protocols were approved by the Ethics Committee of the Turku University Hospital and informed consent was obtained from the guardian before commencing the study.

### Definitions

Atopy was defied as positive IgE antibodies (>0.35 kU/L) for any of the common allergens as previously defined [7,23]. Perennial aeroallergen sensitivity was defined as sensitization (specific IgE >0.35 kU/L) to dog, cat or *Dermatophagoides pteronyssinus*.

### Outcome measures

Pre-defined primary and secondary endpoints were clinical as previously reported [23]. Here, we report the comparison of serum cytokine levels between sole RV or sole RSV (14 other respiratory viruses were ruled out) affected corticosteroid naive wheezing children less than 3 years of age as exploratory endpoints.

### Sample collection and analysis

Laboratory data were collected as previously described [4,7,23,24]. Serum samples were collected on admission before randomization to prednisolone or placebo and 2-3 weeks after discharge. Initially, all available serum samples (75% of eligible) were used according to pre-defined study criteria. The convalescent phase serum samples were analyzed from patients randomized to the placebo group but not from patients randomized to the prednisolone group.

Serum cytokine analyses were done according to manufacturer's instruction by Human Cytokine LINCO *plex *Kit (Millipore Corporation, Billerica, MA). Sensitivity of the kit was as follows (number of samples below detection level of all analysed): interferon (IFN) -gamma 0.29 pg/mL (19/50), interleukin (IL) -2 0.16 pg/mL (18/50), IL-4 0.13 pg/mL (10/50), IL-5 0.01 pg/mL (5/50), IL-10 0.15 pg/mL (0/50), IL-12 0.11 pg/mL (23/50) and IL-13 0.48 pg/mL (24/50). The serum samples were coded, randomly allocated for two batches and laboratory personnel did not know the viral etiology of the cases.

Virus culture was done for adenovirus, influenza A and B viruses, parainfluenza virus (PIV) types 1-3, RSV, enteroviruses, RV and human metapneumovirus (hMPV) [4,24]. Viral antigens were detected for adenovirus, influenza A and B viruses, PIV 1-3 and RSV. Levels of IgG antibodies specific for adenovirus, enteroviruses, influenza A and B viruses, parainfluenza virus types 1/3, RSV were analysed in paired serum samples, in addition to IgM antibodies for enteroviruses. PCR was used for the detection of entero- and RV, RSV, coronaviruses (229E, OC43, NL63 and HKU1), hMPV, human bocavirus, influenza A and B viruses, adenovirus and PIV 1-4. All rhino-enterovirus PCR positive samples could not be typed by hybridization. Twelve such samples, which were available for sequence analysis, all turned out to be RVs. On the basis of this finding, 7 non-typable rhino-enteroviruses were classified as RV. No viral diagnostics were done for the controls.

### Statistics

No statistical power calculation was done for the cytokine analyses. The normality of data distribution was tested using the Kolmogorov-Smirnov test. The t-test, Mann Whitney U test, Chi square test and Spearman's rank correlation were used when appropriate. The cytokine data were analysed using regression analysis (generalized linear model with binomial distribution and log-link). The backward stepwise multivariate analysis of the differences in cytokine levels between RV and RSV infection was adjusted to age, presence of atopy, days of preceding cough, blood eosinophil count and presence of acute otitis media (i.e. the baseline differences). Only significant adjustments, i.e. *P *< 0.05, were kept in the model. The cytokine data is presented as a fold-difference between RV and RSV affected children. The statistical analyses were carried out using SAS/STAT(r) software, Version 9.1.3 SP4 of the SAS System for Windows, SAS Institute Inc., Cary, NC, USA.

## Results

### Characteristics of study children

During the study period, 661 children were hospitalized for acute wheezing. Of these, 293 were enrolled in the VINKU-study (Fig. 1). Of the 293 children, 67 children fulfilled study criteria, but in 17 cases (11 RV and 6 RSV positive) serum samples were not available. The 50 children included were 3 to 34 months old, had their first or second wheezing episode, had confirmed sole RV or sole RSV infection (14 other respiratory viruses were ruled out) and were corticosteroid naive at study entry. Twenty-three children were affected by RV and 27 by RSV. Of the 23 RV positive cases, 19 [83%] were positive by PCR and 4 [17%] by culture, and of the 27 RSV cases, 26 [96%] were positive by culture, 26 [96%] by antigen detection, 23 [85%] by PCR and 18 [67%] by serology. The demographics of age (p > 0.1), sex (p > 0.5), atopy (p > 0.3) and blood eosinophil count (p > 0.6) did not differ between the eligible children with sera available (n = 50) compared to those without (n = 17) nor in the subgroups of RV (n = 34) and RSV (n = 33) positive children (respectively, p > 0.1 and p > 0.3 for all comparisons within virus groups).

The children positive for RV were slightly older and had more pronounced atopic/airway inflammatory characteristics, i.e. levels of allergic sensitization to foods, blood eosinophils, and exhaled nitric oxide, than those positive for RSV (Table 1). The longer duration of preceding respiratory symptoms, acute otitis media and use of antibiotics were more common in the RSV-group than in the RV-group. No other differences were found in the baseline characteristics.

**Table 1:** Patient characteristics in rhinovirus and respiratory syncytial virus affected children.

	Rhinovirus (n = 23)	RSV (n = 27)	*P*
Age, years	1.4 (0.59)	0.78 (0.61)	0.0012
Male, No.	15 (65%)	16 (59%)	0.67
Atopic, No.	10 (44%)	1 (4%)	0.0009
Food sensitization, No	9 (39%)	1 (4%)	0.0034
Perennial aeroallergen, No.	1 (4%)	0 (0%)	0.47
1^st ^episode, No.	17 (74%)	25 (93%)	0.12
2^nd ^episode, No.	6 (26%)	2 (7%)	0.12
Parental asthma, No.	2 (9%)	6 (22%)	0.26
Parental allergy, No.	13 (57%)	16 (59%)	0.85
Parental smoking, No.	11 (48%)	14 (52%)	0.78
			
Previous symptoms			
Cough, days	2 (1, 3)	4 (3, 5)	0.0072
Wheezing, days	1 (1, 1)	2 (2, 3)	0.053
			
On entry to the study			
RSS, points (0, none to 12, severe)	6.7 (1.4)	6.5 (1.1)	0.45
O_2_-saturation, %	96 (2.3)	96 (2.5)	0.75
Acute otitis media, No.	11 (48%)	21 (78%)	0.028
Blood eosinophils, × 10^9^/L	0.4 (0.2, 0.6)	0.0 (0.0, 0.1)	<0.0001
Blood eosinophils >0.1 × 10^9^/L	20 (87%)	3/26 (12%)	<0.0001
Exhaled nitric oxide, ppb^1^	7.5 (7.0, 12)	5.1 (3.9, 6.5)	0.020
			
Medication			
Salbutamol at ER before entry, mg/kg	0.23 (0.18)	0.19 (0.17)	0.46
Antibiotic treatment, No.	15 (65%)	21 (78%)	0.013

RSV, respiratory syncytial virus; RSS, respiratory symptom score; ER, emergency room.

Values are means (SD), No. (%), or medians (interquartile range).

Continuous data were analysed by t-test except Mann Whitney U test was used for blood eosinophil and exhaled nitric oxide levels. Categoric data were analysed by Chi square test.

^1^rhinovirus group, n = 11, RSV-group, n = 17.

Eleven children without respiratory symptoms served as asymptomatic controls for the RV-group. The demographics of this control group (mean age 1.0 year [sd 9 months]; 5/11 [45%] male; 6/11 [55%] atopic; and median blood eosinophil count 0.26 × 10^9^/L [interquartile range 0.11, 0.54]) did not differ from the RV-group (respectively, p > 0.1, p > 0.4, p > 0.5 and p > 0.3).

### Cytokine levels at acute phase

The RV affected children had higher IL-13, IL-12, IFN-gamma and IL-5 concentrations in serum than those affected by RSV at acute phase in univariate models (n = 50, Tables 2 and 3). All these differences remained relatively stable in multivariate models which showed that the RV-group had 39-fold higher IL-13 (p < 0.0001), 7.5-fold higher IL-12 (p < 0.0001), 6.0-fold higher IFN-gamma (p = 0.0005) and 2.8-fold higher IL-5 (p = 0.021) concentrations in serum than the RSV group. When compared to the asymptomatic control group, the RV-group had 27-fold higher IFN-gamma (p < 0.0001), 8.9-fold higher IL-2 (p = 0.0007), 5.6-fold higher IL-5 (p = 0.0018) and 2.6-fold higher IL-10 (p = 0.0098) serum concentrations at acute phase (n = 34, Tables 2 and 3).

**Table 2:** Serum cytokine levels in rhinovirus and respiratory syncytial virus associated acute wheezing and in the asymptomatic age and atopy matched control group for rhinovirus group.

Cytokine	Rhinovirus associated acute wheezing		Respiratory syncytial virus associated acute wheezing		Asymptomatic age and atopy matched control group for rhinovirus group	
			
	n	median (interquartile range)	n	median (interquartile range)	n	median (interquartile range)
Th_1_-type						
IFN-gamma	23	12 (9.3, 58)	27	0.00 (0.00, 3.6)	11	0.00 (0.00, 0.00)
IL-2	23	7.87 (2.0, 14)	27	0.00 (0.00, 1.1)	11	0.00 (0.00, 0.82)
IL-12	23	6.5 (0.00, 16)	27	0.00 (0.00, 1.5)	11	0.00 (0.00, 0.00)
						
Th_2_-type						
IL-4	23	120 (52, 190)	27	17 (0.00, 130)	11	63 (6.5, 460)
IL-5	23	7.4 (2.6, 17)	27	0.50 (0.12, 3.0)	11	1.8 (0.00, 3.8)
IL-13	23	53 (17, 77)	27	0.00 (0.00, 0.86)	11	26 (14, 34)
						
T_reg_-type						
IL-10	23	43 (28, 74)	26	83 (39, 140)	11	25 (14, 34)

CI, confidence interval; Th, T-helper cell; IFN, interferon; IL, interleukin; T_reg_, T-regulatory cell.

The unit for all cytokine levels is pg/mL.

**Table 3:** Difference in cytokine levels when rhinovirus associated acute wheezing is compared to respiratory syncytial virus associated acute wheezing and to asymptomatic age and atopy matched control group.

Cytokine	RV-group compared to RSV-group						RV-group compared to control group		
				
	Univariate			Multivariate					
			
	n	fold difference (95% CI)^1^	*P*	fold difference (95% CI)	*P*	adjustments	n	fold difference (95% CI)	*P*
Th_1_-type									
IFN-gamma	50	6.0 (2.1, 18)	0.0005	6.0 (2.1, 18)	0.0005	-	34	27 (7.6, 88)	<0.0001
IL-2	50	0.51 (0.13, 2.0)	0.30	0.51 (0.13, 2.0)	0.30	-	34	8.9 (2.9, 26)	0.0007
IL-12	50	11 (3.9, 29)	<0.0001	7.5 (2.6, 21)	<0.0001	age	34	1.1 (0.16, 5.3)	0.92
									
Th_2_-type									
IL-4	50	0.90 (0.32, 2.5)	0.84	0.90 (0.32, 2.5)	0.84	-	34	1.0 (0.39, 2.4)	0.98
IL-5	50	2.8 (1.1, 7.0)	0.021	2.8 (1.1, 7.0)	0.021	-	34	5.6 (2.1, 14)	0.0018
IL-13	50	39 (14, 110)	<0.0001	39 (14, 110)	<0.0001	-	34	0.40 (0.14, 1.0)	0.061
									
T_reg_-type									
IL-10	49	0.59 (0.33, 1.1)	0.066	0.72 (0.40, 1.3)	0.27	atopy	34	2.6 (1.3, 4.9)	0.0098

RV, rhinovirus; RSV, respiratory syncytial virus; CI, confidence interval; Th, T-helper cell; IFN, interferon; IL, interleukin; T_reg_, T-regulatory cell.

The data were analysed using regression analysis. In multivariate analysis, adjustments to age, presence of atopy, days of preceding cough, blood eosinophil count and presence of acute otitis media (i.e. the baseline differences) were used in a backward stepwise model. Only significant adjustments (*P *< 0.05) were kept in the model.

^1^i.e. how many folds higher cytokine level is in the RV-group than in the RSV-group or in the control group.

### Cytokine levels at convalescence phase

Two to three weeks after discharge (n = 19), the RV affected children had higher IFN-gamma, IL-13, IL-12, IL-2, IL-10, IL-12 and IL-2 concentrations in serum than the RSV affected children in univariate models (Tables 4 and 5). In multivariate models, the RV-group had >100-fold higher IFN-gamma (p = 0.0090), 33-fold higher IL-13 (p < 0.0001) and 6.5-fold higher IL-10 (p < 0.0001) concentrations in serum than the RSV-group. The differences in IL-12 and IL-2 did not persist after adjustments for the baseline differences. When compared to the asymptomatic control group, the RV-group had 15-fold higher IFN-gamma (p = 0.010) and 9.4-fold higher IL-2 (p = 0.0048) serum concentrations in the convalescence phase (n = 20, Tables 2 and 4).

**Table 4:** Difference in cytokine levels in corticosteroid naive children 2-3 weeks after hospitalization for wheezing when rhinovirus associated acute wheezing is compared to respiratory syncytial virus associated acute wheezing wheezing and to asymptomatic age and atopy matched control group.

Cytokine	RV-group compared to RSV-group						RV-group compared to control group		
				
	Univariate			Multivariate					
			
	n	fold difference (95% CI)^1^	*P*	fold difference (95% CI)	*P*	adjustments	n	fold difference (95% CI)	*P*
Th_1_-type									
IFN-gamma	19	49 (37, 170)	0.0003	120 (6.5, 45000)	0.0090	atopy, eos	20	15 (1.4, 210)	0.010
IL-2	19	8.7 (2.2, 36)	0.0012	1.2 (0.16, 6.3)	0.84	atopy, age, eos	20	9.4 (1.7, 56)	0.0048
IL-12	19	17 (2.4, 110)	0.0014	0.72 (0.047, 14)	0.80	eos	20	1.4 (0.07, 39)	0.80
									
Th_2_-type									
IL-4	19	0.81 (0.13, 5.2)	0.80	0.61 (0.11, 4.0)	0.56	age, atopy	20	1.0 (0.21, 5.4)	0.98
IL-5	19	1.6 (0.37, 6.8)	0.51	1.6 (0.37, 6.8)	0.51	-	20	2.5 (0.82, 8.2)	0.086
IL-13	19	33 (5.5, 190)	<0.0001	33 (5.5, 190)	<0.0001	-	20	0.40 (0.11, 1.6)	0.16
									
T_reg_-type									
IL-10	19	7.7 (2.7, 22)	<0.0001	6.5 (2.7, 16)	<0.0001	otitis	20	2.6 (1.3, 4.9)	0.11

RV, rhinovirus; RSV, respiratory syncytial virus; CI, confidence interval; Th, T-helper cell; IFN, interferon; eos, blood eosinophil count; IL, interleukin; T_reg_, T-regulatory cell.

The data were analysed using regression analysis. In multivariate analysis, adjustments to age, presence of atopy, days of preceding cough, blood eosinophil count and presence of acute otitis media (i.e. the baseline differences) were used in a backward stepwise model. Only significant adjustments (*P *< 0.05) were kept in the model.

^1^i.e. how many folds higher cytokine level is in the RV-group than in the RSV-group or in the control group.

**Table 5:** Differences in serum cytokine levels in acute and convalescence phases of rhinovirus and respiratory syncytial virus associated early wheezing episodes in corticosteroid naive children.

Cytokine	Rhinovirus					RSV					Comparison of differences
			
	n	Acute	Convalescence	Difference	*P*	n	Acute	Convalescence	Difference	*P*	*P*
Th_1_-type											
IFN-gamma	9	32 (11, 60)	10 (1.1, 49)	-2.3 (-9.8, 8.6)	0.28	10	0.00 (0.00, 2.7)	0.00 (0.00, 0.00)	0.00 (-2.7, 0.00)	0.33	0.90
IL-2	9	9.99 (2.3, 13)	4.5 (0.83, 20)	-2.3 (-6.9, 2.6)	0.59	10	0.28 (0.00, 1.7)	0.70 (0.00, 2.8)	0.00 (-0.53, 0.28)	0.51	0.44
IL-12	9	13 (0.30, 16)	3.5 (0.00, 16)	-0.30 (-1.4, 3.5)	0.86	10	0.00 (0.00, 0.41)	0.00 (0.00, 2.2)	0.00 (-0.24, 0.00)	0.83	0.54
											
Th_2_-type											
IL-4	9	190 (120, 360)	120 (34, 280)	-28 (-68, 9.5)	0.16	10	16 (0.00, 65)	43 (6.5, 150)	12 (0.00, 55)	0.34	0.037
IL-5	9	14 (7.4, 28)	3.5 (2.0, 14)	-12 (-14, -1.3)	0.16	10	0.41 (0.08, 1.0)	0.60 (0.20, 0.88)	0.13 (-0.15, 0.68)	0.60	0.046
IL-13	9	59 (25, 81)	52 (13, 70)	-7.4 (-12, 43)	0.93	10	0.00 (0.00, 3.8)	0.00 (0.00, 3.8)	0.00 (0.00, 0.00)	0.86	0.59
											
T_reg_-type											
IL-10	9	75 (52, 120)	32 (11, 57)	-44 (-64, -22)	0.039	10	54 (39, 129)	9.1 (4.1, 14)	-50 (-121, -25)	0.0005	0.66

CI, confidence interval; Th, T-helper cell; IFN, interferon; IL, interleukin; T_reg_, T-regulatory cell.

Values are medians (interquartile range). Data were analysed by Mann Whitney U test.

The unit for all cytokine levels is pg/mL.

### Cytokine levels: acute vs. convalescence

Nineteen corticosteroid naive wheezing children had both acute and convalescent samples available. Serum IL-10 levels were higher in acute phase compared to convalescence phase both in RV (p = 0.039) and RSV infections (p = 0.0005, Table 5). At acute phase, IL-10 levels correlated strongly and positively with other cytokines in RV affected children and only with IFN-gamma in RSV affected children (Table 6). Overall, IL-10 did not correlate with age (r = 0.04, p = 0.76).

**Table 6:** Correlation between serum IL-10 and other cytokine levels in corticosteroid naive children with acute wheezing.

Cytokine	Rhinovirus				Respiratory syncytial virus			
		
	n	acute	n	convalescence	n	acute	n	convalescence
Th_1_-type								
IFN-gamma	23	r = 0.57	9	r = 0.63	26	r = 0.43	10	r = 0.52
		*P *= 0.0047		*P *= 0.069		*P *= 0.029		*P *= 0.12
IL-2	23	r = 0.50	9	r = 0.63	26	r = -0.27	10	r = 0.34
		*P *= 0.016		*P *= 0.069		*P *= 0.19		*P *= 0.34
IL-12	23	r = 0.54	9	r = 0.43	26	r = -0.027	10	r = 0.66
		*P *= 0.0074		*P *= 0.24		*P *= 0.90		*P *= 0.040
								
Th_2_-type								
IL-4	23	r = 0.44	9	r = 0.58	26	r = 0.084	10	r = 0.42
		*P *= 0.035		*P *= 0.10		*P *= 0.68		*P *= 0.23
IL-5	23	r = 0.53	9	r = 0.33	26	r = -0.097	10	r = 0.0061
		*P *= 0.0096		*P *= 0.39		*P *= 0.64		*P *= 0.99
IL-13	23	r = 0.50	9	r = 0.50	26	r = 0.11	10	r = 0.55
		*P *= 0.014		*P *= 0.17		*P *= 0.58		*P *= 0.10

CI, confidence interval; Th, T-helper cell; IFN, interferon; IL, interleukin.

Data were analysed by Spearman's rank correlation.

Next, we compared differences between acute and convalescence phases between RV and RSV infections. Such difference were found in IL-4 (p = 0.037) and IL-5 (p = 0.046) levels (Table 5). The levels of these cytokines non-signifantly decreased in RV-group whereas they non-significantly increased in RSV-group.

### Analysis of bias

Since 17 eligible children did not have sera available, we tested if it could bias the results. The missing values in RSV cases were corrected as upper 95% confidence interval values and the missing values in RV cases were corrected as lower 95% confidence interval values of corresponding cytokines, and the difference in cytokine levels in all eligible children during acute wheezing was analysed. In this extreme supplementary analysis, the RV-group had 16-fold higher IL-13 (p < 0.0001), 4.8-fold higher IL-12 (p = 0.0015), and 2.4-fold higher IFN-gamma (p = 0.098) serum concentrations than RSV-group suggesting that the direction of the difference of these cytokines is true (otherwise data not shown).

## Discussion

Cytokine dysregulation, Th_1_/Th_2 _imbalance, plays an important role in the development of asthma and allergic diseases. At birth and later in early life, blood cytokine profile indicates whether PBMC response is skewed toward a Th_2_-phenotype (production of IL-4, IL-5 and IL-13) and away from Th_1_-phenotype (production of IFN-gamma). The relative nature of this Th_1_/Th_2 _imbalance, especially IFN-response early in life, has been linked to antiviral activity and the subsequent development of allergic disease and/or asthma [25].

The principal Th_1_-cytokine, IFN-gamma, is likely to be most important cytokine responsible for cell mediated immunity [25]. It is primarily produced by T-helper lymphocytes but is also derived from cytotoxic T cells and NK cells. IFN-gamma production belongs to nonspecific defense mechanims, which have direct immunoregulatory and antiviral actions, although its capability to inhibit viral replication is modest. IFN-gamma also inhibits allergic responses through its capacity to inhibit IL-4 mediated effects but may also contribute to airway hyperresponsiveness especially in non-atopic subjects [26]. Previous *in vitro *and *in vivo *studies in adults have shown that IFN-gamma is highly and dose-dependently inducible by RV in leukocyte, T cell, or airway epithelial cell cultures and that blood CD4^+ ^IFN-gamma response is associated with lower RV loads and less severe symptoms suggesting protective role for IFN-gamma [11,15,17]. Our study further suggests an important role for the IFN-gamma response in RV associated early wheezing. The results from the comparison between RV-group and the control group argue against the suggestion that the high IFN-gamma levels in RV affected children were due to predisposition. Previous findings in murine models and our findings also support the link between IL-12 and IFN-gamma in virus infection, i.e. the former induces the latter [27]. However, the role of IL-2 and IL-12 appears to be less pronounced in RV infections as shown by us here and by others previously [11,15]. IFN-gamma response was low in RSV affected children as expected [27,28].

Th_1_/Th_2 _cytokine ratio seems to determine the course of clinical illness, i.e. those with lower ratio have more severe illness [12,13,16,17]. The capability of RV to induce Th_2_-type cytokines (IL-4 and IL-5) has been less pronounced in previous *in vitro *studies compared to IFN-gamma responses as also supported by *in vivo *data [14,29]. Although it is possible that RV-infection worsens Th_2_-type inflammation, the presence of Th_2_-type cytokines more likely may reflect a chronic inflammatory state of lower airways, which may increase susceptibility to RV infections [17,30]. Furthermore, Th_2_-type cytokines could counteract Th_1_-type cytokines, and thereby, may increase susceptibility to more severe RV-infections [12,15,17,25]. In agreement, the RV affected children had markedly higher systemic levels of IL-5 and IL-13 than those affected by RSV in our study, but this difference appeared to be linked to atopy. The difference in Th_2_-type cytokines was not so striking when RV-group was compared to atopy/age-matched controls and seen only in IL-5 in the acute phase and not seen at all in the convalescence phase. These findings fit in the previously reported close association between RV infections and atopic characteristics [5,14]. Interestingly, the IL-13 response was markedly higher in RV affected children than in those affected by RSV in our study. IL-13 is known to play critical role in airway hyperreactivity and amplifying allergic inflammation in asthma [17,26]. It should be noted that certain polymorphism, such as IL-4 590T and IL-4Ralpha R551 alleles, could be linked to more sereve bronchiolitis and the IL-13 Gln allele may identify children at risk for persistent wheezing as shown in RSV affected children [18,20].

Of the studied cytokines, IL-10 tends to have immunoregulatory properties and its generation is usually associated with resolution of the inflammatory process [31]. Moreover, it is also associated with airway hyperreactivity, and blood CD 4^+ ^IL-10 level has inversely correlated with nasal RV load [17,26]. Our findings on IL-10 are very similar to the reports by Grissell et al. (2005) [32]. They studied cytokine gene expression by quantitative PCR in the induced sputum of mainly adult subjects (>7-year-old) with virus induced acute asthma (64% of subjects had RV). They found that IL-10 mRNA was increased in virus-infected acute asthma and reduced on recovery phase. We further showed that IL-10 was elevated in both virus groups in the acute phase when compared to the convalescence phase and also when RV-group was compared to the control group. The finding that IL-10 level was greater in the RV-group than in the RSV-group in the convalescent phase is probably linked to predisposition (atopy) since no difference was found in this time-point when compared to the control group. The increased IL-10 level in acute viral infection could mean that IL-10 is a causative mediator in virus provoked exacerbation (triggered by IFN-gamma), and that the generation of IL-10 is a response to a greater degree of pre-existing airway inflammation in individuals predisposed to virus, especially to RV, induced exacerbations, and serve to promote tolerance [17,33]. Whether IL-10 levels reflect T_reg _activity, it is intriguing to speculate that the patients with decreased IL-10 responses (thereby defective down regulation of Th_1 _and Th_2 _responses) could have greater inflammatory response to viral infections (e.g. to >150 circulating RV strains) [9,10] and thereby increased risk for recurrent wheezing. In agreement, a recent study reported lower IL-10 levels in stimulated cord blood of children who were hospitalized for RSV infection before 6 months of age compared to those who were treated as outpatients [34]. Interestingly, children homozygous for the IL-10 -592C, -592A or IL-10 -1082A allele have had a high risk of severe bronchiolitis [19,21].

It can be debated whether rhinovirus PCR positivity can be considered as an indication of a real infection [3]. Recent data, however, suggests that it can. First, RV-PCR positive respiratory findings have been linked to the severity of respiratory illness [1,35]. Second, although virus replication lasts usually longer than clinical illness, PCR positivity has been rather short-lasting (usually <2 weeks) in RV-genotype specific analysis [35]. Third, the current data adds that RV PCR positivity is also linked to systemic immune responses.

The strengths of the study include detailed viral diagnostics, careful characterization of atopic characteristics, natural illness and *in vivo *samples of normal population although our hospitalized cohort probably represents the most severe end of illness. The group of corticosteroid naive children in the follow-up was not biased since all the study children were initially randomized to prednisolone or placebo treatments. The use of non-steroidal anti-inflammatory drugs or acetaminophen was not recorded, but their effect on cytokine levels is considered negligible when recommended doses are used [36,37] As a limitation, newly recognized type-C and type-D RVs may have evaded our PCR [9,10]. Furthermore, systemic cytokine responses may not reflect the responses in the airways [38].

The study demonstrates the difficulty of comparing RSV- and RV affected young wheezing children since RV infection is closely associated with atopy, whereas RSV is not, and RV typically affects slightly older children than RSV infection (5, 22). Almost 2 year recruitment period in a relatively large university hospital resulted only 2 RV cases of all eligible cases to match RSV cases (1^st ^episode, non-topic and blood eosinophil count <0.4 × 10^9^/L). On the other hand, RSV cases are difficult to match to RV cases due to rare association to atopy (only one case in our study).

## Conclusion

The young wheezing children infected by RV and RSV differ significantly in terms of age, atopic characteristics, or systemic cytokine responses. Our results support a hypothesis that rhinovirus is likely to trigger early wheezing in children with a predisposition, who are old enough to develop a distinct atopy-related asthma-prone phenotype [5]. The elevated levels of IFN-gamma, IL-13 and IL-10 (pre-existing or virus-induced) in RV-affected children underline the importance of these cytokines in the early pathogenesis of asthma and the detection of RV as an important, maybe not a risk factor, but a revealing factor for a high risk phenotype [5-8,17,26].

## Abbreviations

RV: rhinovirus; RSV: respiratory syncytial virus; RNA: ribonucleic acid; Th: T-helper cell; T_reg_: T-regulatory cell; IgE: immunoglobulin E; hMPV: human metapneumovirus; PIV: parainfluenza virus; PCR: polymerase chain reaction; RSS: respiratory symptom score; sd: standard deviation; CI: confidence interval; IL: interleukin; IFN: interferon.

## Declaration of competing interests

The authors declare that they have no competing interests.

## Authors' contributions

TJ designed the VINKU study with OR, recruited and followed clinically half of the patients, performed the statistical analyses and drafted the manuscript. MP-A carried out the molecular studies. PL recruited and followed clinically half of the patients, participated in coordination and helped to draft the manuscript. VP carried out the molecular studies and helped to draft the manuscript. TV was in charge of the viral studies. OS was in charge of the molecular studies, and helped to draft the manuscript. OR designed the VINKU study with TJ, and was in charge of the clinical studies, and helped to draft the manuscript. All authors read and approved the final manuscript.
